# Anchorage independence altered vasculogenic phenotype of melanoma cells through downregulation in aminopeptidase N /syndecan-1/integrin β4 axis

**DOI:** 10.18632/aging.103425

**Published:** 2020-08-04

**Authors:** Yu-Che Cheng, Wei-Chi Ku, Ting-Ting Tseng, Ching-Po Wu, Mengjin Li, Shao-Chen Lee

**Affiliations:** 1 School of Medicine, College of Medicine, Fu Jen Catholic University, New Taipei, Taiwan; 2 Proteomics Laboratory, Cathay Medical Research Institute, Cathay General Hospital, Taipei, Taiwan; 3 Department of Biomedical Science and Engineering, National Central University, Jhongli, Taiwan

**Keywords:** anchorage independence, melanoma, integrin β4, aminopeptidase N, syndecan-1

## Abstract

The detachment of tumor cells from extracellular matrix and survival under anchorage-independence were recognized as the initial step of tumor metastasis. Previously we had demonstrated that anchorage-independence altered gene expressions and showed characteristics of cell invasiveness loss, enhanced chemosensitivity, and enhanced subcutaneous tumor formation. However, whether it affected histological phenotypes in tumor tissues remained unclear.

Melanoma metastases were generated in nude mice using adherent or suspended melanoma cells. Examination of melanoma metastases revealed histological features of extensive vascular structures in adherent cell-derived tumors, while not seen in suspended cell-derived tumors. Quantitative proteomic analysis at adherent, suspended, and re-attached melanoma cells suggested that aminopeptidase N was potentially downregulated upon cell suspension or reattachment. Downregulation of aminopeptidase N by gene-specific shRNAs showed reduced cell invasiveness and enhanced subcutaneous tumor formation that was consistent with previous observations. Experiments by suppression or overexpression of aminopeptidase N expression demonstrated that aminopeptidase N regulated syndecan-1 and integrin β4 expression through PKCδ pathway. Histological analysis at melanoma metastases further suggested that CD31^+^/aminopeptidase N^+^/syndecan-1^+^/integrin β4^+^ phenotypes were associated with vascular structures.

In summary, we suggested the expression axis of aminopeptidase N/syndecan-1/integrin β4 in melanoma cells was suppressed by detachment stress, which diminished vascular phenotypes of melanoma metastases.

## INTRODUCTION

Metastatic melanoma is one of the aggressive types of cancer with high morbidity, high mortality, and poor prognosis. Metastasis of tumor cells could be divided into several stages, including detachment from primary site, intravasation into the circulation system, dissemination in anchorage-independent manner, and lodging at the secondary site with successive angiogenesis process. It was shown that gene alteration by melanoma metastasis contributed to their diverse phenotypes and altered tissue heterogeneity. Those could explain the tumor complication and resistance to conventional therapy. To understand and reveal the potential mechanisms connecting the genetic and biological heterogeneity were of critical importance.

Anchorage-independent survival is one of specific features for malignant tumors, including melanoma. Detachment of tumor cells from surroundings is the initial step of premetastatic process, so that malignant melanoma cells survive under stressed condition. Previously, we had demonstrated that anchorage-independence altered the phenotypes and characteristics of melanoma cells. Anchorage impairments led to the loss of invasiveness that were driven by PKCδ-mediated downregulation of syndecan-1 (SDC1), reduced expression of matrix metalloprotease-2 (MMP2) protein, and reduced laminin-binding ability [1]. However, the chemosensitivity of melanoma cells was increased by suspension culture that upregulated syndecan-2 (SDC2) protein expression by PI3K and ERK pathway activation [2]. This was also associated with elevated PKCδ activation. In addition, the subcutaneous tumor growths were promoted by anchorage independence through the mechanism of increased IL-8 protein secretion and upregulation of IL-8 receptor CXCR1, which were contributed by ERK and JNK activation [3].

SDC1 is one of syndecan family proteins classified as the transmembrane-type heparan sulfate-proteoglycan. It participated in several cellular functions such as cell growth, differentiation, adhesion, or migration. Those cell activities were potentially mediated though extracellular ligands binding at glycosaminoglycan chains thus activated intracellular signaling [4–7]. SDC1 also promotes tumor growth, angiogenesis, and invasive phenotype in many tumor models [8, 9]. Angiogenesis is a critical event in the development and dissemination of human tumors. Another membrane protein family, integrin, also participates in blood and lymphatic vessel growth by promoting endothelial cell migration and survival [10–12]. However, the evidences to show the regulation and connection between these proteins need to be addressed.

In this study, we found that anchorage-independence of melanoma cells downregulated aminopeptidase N (ANPEP) expression, which downregulated SDC1 expression, thus further downregulated integrin β4 expression. The change in ANPEP-SDC1-integrin β4 expression axis reduced to angiogenetic ability of melanoma cells. This explained the detachment stress would the one of primary factors affecting the presence or absence of vascular structures in melanoma.

## RESULTS

### Loss of vascular structure in the tumor derived from suspended melanoma

**Figure 1:**
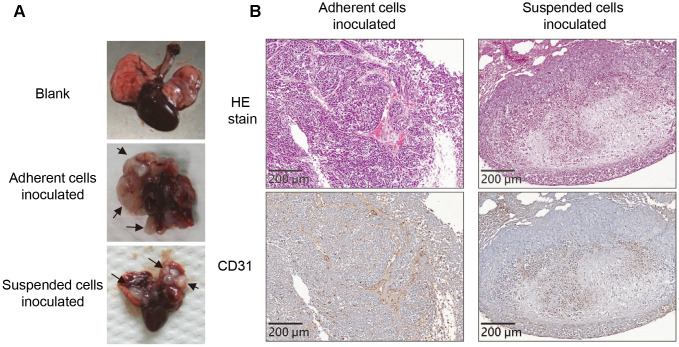
**Loss of vasculogenic phenotype in the metastatic nodules formed by suspended melanoma cells.** (**A**) Several white nodules were developed in the lungs of mice injected with either adherent or suspended melanoma cells. (**B**) HE staining and CD31 immunostaining showed vascular structures in the tumor nodules derived from adherent melanoma cells, but not seen in those from suspended melanoma cells.

We established the lung metastases of adherent or suspended melanoma cells, and examined the xenograft tumor samples to see whether detachment stress played roles in different phenotypes. These were achieved by injection of the adherent or suspended melanoma cells into tail veins of nude mice [13, 14]. Tumor disseminated right away in the circulation that would simulate the post-intravasation stage and allowed cell-trapping at lung tissues for the development of lung metastasis [13–15]. Two groups of nude mice were inoculated with either adherent or suspended melanoma cells and allowed tumor development for 2 months. Upon inspection of the lungs of these mice inoculated with either adherent or suspended melanoma cells, multiple white nodules were grossly developed as metastases derived from amelanotic cells (Figure 1A). No significant difference in the number or the size of nodules in the lung tissues of these two groups of mice.

Pathological dissection with HE stain showed totally different architectures in the nodules generated by adherent or suspended melanoma inoculations. As shown in Figure 1B, networks of connective tissues were both observed in the nodules derived adherent or suspended melanoma cells. In the nodules derived from adherent melanoma cells, dominant vascular tubes infiltrated with red blood cells (RBCs) were seen. These tubular structures of hyaline cavities were embedded and surrounded by loose connective tissues with diluted fibrous structures. These nodule tissues were also immunostained to visualize the endothelial cell marker of the vessel, CD31. It showed that most of RBC-containing cavities were CD31-positive. It indicated the generation of vascular structure in the neoplastic nodules derived from adherent melanoma cells. For the nodules derived from suspended melanoma cells, no dominant vascular cavities were seen. However, some RBCs were infiltrated into the loose connective networks. As to CD31 immunostaining, no clear vascular structures were observed in the nodules derived from suspended melanoma cells. This suggested the loss of vasculogenic phenotypes for melanoma cells after suspension treatment.

### Proteomic analysis showed downregulation of ANPEP in suspended melanoma cells

In order to find out the potential targets correlated with the loss of vascular structures in nodules derived suspended melanoma cells, we compared the protein contents in the adherent, suspended, and re-attached melanoma cells by proteomic analysis. The tumor metastases from suspended melanoma cells could be recognized as re-attachment of suspended melanoma cells.

In order to increase the peptide identification, we divided protein lysate as cytosolic and nuclear protein fractions. After protein digestion by Lys-C/trypsin, and followed dimethyl labeling, each part were further separated into 5 fractions by strong cation exchanger. They were analyzed using LTQ Orbitrap mass spectrometer connected with the HPLC system. The peptide targets from each fraction were integrated and their protein identities were determined by the mass of fragmented peptides compared using SwissProt database.

Several protein identities were upregulated or downregulated in suspended melanoma cells or re-attached melanoma cells comparing to those in adherent cells (Table 1). Totally 61 proteins were significantly changed upon cell suspension with 27 protein upregulated and 34 protein downregulated. Totally 97 proteins were significantly changed upon cell reattachment after suspension with 44 protein upregulated and 53 protein downregulated.

**Table 1:** Significantly changed proteins in melanoma cells under suspension or re-attachment.

**Cells**	**Expression**	**Protein identity in gene symbol**
Suspension vs adhesion	Upregulated	APIP, TOX1, CAPS, CARHSP1, EIF4EBP1, GALNT2, GPATCH1, HLA-DRB1, IL13RA2, **JMJD6**, LGALS3, MAGEC2, MCM6, NES, PAPSS2, POP5, PSAP, RCN1, RNMT, S100A1, S100B, SYNM, TAGLN3, TBC1D15, TIMELESS, TRMT61A, VIM
Suspension vs adhesion	Downregulated	ABCD3, AHNAK, AKR1B1, **ANPEP**, **ANXA2**, ANXA6, ATP5D, BCAR3, CALB2, **CAV1**, CCDC101, CDC27, CNN3, **CSPG4**, CSTF2T, CT45A3, CTNND1, EHD2, EIF4H, EPHA2, FBLL1, FLNA, FLNB, GNE, GPRC5A, GTF2H4, ITGB4, KANK3, LAMB1, LASP1, NCAPH2, NNMT, PDLIM1, PRKCDBP, PTRF, RBM42, SLC2A1, SLC3A2, SLC7A5, SLC9A3R2, SMC1B, STT3B, SUPT16H, **TGM2**
		
Re-attachment vs adhesion	Upregulated	AGFG1, ANAPC5, ARMC6, ATOX1, BAZ2A, CAPS, CSRP2, CTSL, CTSZ, DSTN, ERP44, GALNT2, HLA-DRB1, IL13RA2, INPP5F, LEPRE1, LGALS3, NES, PAPSS2, POLD3, PRIM2, PSAP, RNMT, S100A1, S100B, SCARB1, SLC12A2, SLC1A4, SPARC, STT3B, SYNM, TAGLN3, VIM, ZNF593
Re-attachment vs adhesion	Downregulated	ADRM1, AHNAK, AKR1B1, **ANPEP**, ANXA6, BCAS2, CALB2, **CAV1**, CCDC101, CDC27, CETN2, **CHD7**, DDB2, DMPK, DPYSL3, EHD2, FAM129B, FBXO22, FBXO28, FLNB, GDA, GNE, GPRC5A, GRPEL2, HIST1H2BL, HIST1H4A, HIST2H3A, HNRNPA3, INTS12, ITGA6, ITGB4, KANK3, KIF24, LASP1, LEPREL4, MLF2, NFYA, NIFK, NNMT, NR2C2, PDLIM1, POLE3, PSPC1, PTPN23, PTRF, SAMHD1, SCD, SLC2A1, SMC1B, STK3, **TGM2**, TNS3, TUBB8

The gene products which involved in blood vessel formation were shown in bold.

In further, we used the webtool DAVID (The Database for Annotation, Visualization and Integrated Discovery [16, 17]) to analyze the changed proteins to associate with the loss of vascular structures. Functional annotations showed enriched clusters at different terms of gene ontology (biological process, cellular component, and molecular function).

**Figure 2:**
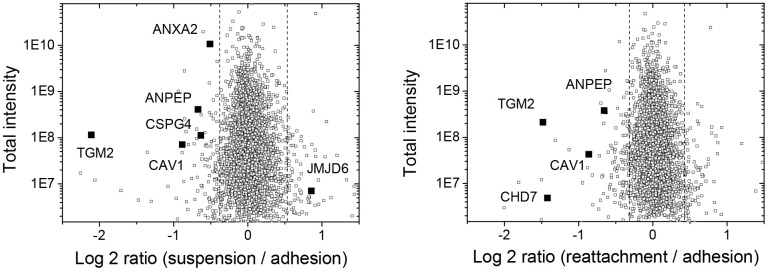
**Plots of log2-ratio and intensity to show the distribution of upregulated and downregulated protein identities in suspended melanoma and re-attached melanoma cells as analyzed by quantitative proteomic analysis.** The significantly changed proteins associated with vessel development were indicated by solid squares.

As shown in Tables 2 and 3, the changes in protein expression were categorized and mostly enriched at nucleus for the processing of nucleic acid synthesis. The molecular function associated with blood vessel formation or angiogenesis was also highlighted, which was correlated with our observation (Figure 1). As showing in Table 1 and Figure 2, there were 5 downregulated proteins (ANPEP; aminopeptidase N, ANXA2; annexin A2, CAV1; caveolin-1, CSPG4; chondroitin sulfate proteoglycan 4, and TGM2; transglutaminase 2) associated with blood vessel/vasculature development in suspended melanoma cells; while 4 downregulated proteins (ANPEP, CAV1, CHD7; Chromodomain-helicase-DNA-binding protein 7, and TGM2) associated with blood vessel/vasculature development in re-attached melanoma cells. Accordingly, it suggested that these 3 downregulated proteins (ANPEP, CAV1, and TGM2) were potentially remained downregulated and might associate with the loss of vascular structures in tumor metastases. Comparing our previous microarray data (GSE42876 and GSE61671) with the result of current proteomic analysis, the positive and strong correlation that both *ANPEP* and *CAV1* gene products were downregulated in suspended melanoma cells and reattached melanoma cells. No significant change was seen in the result of cDNA microarray analysis for *TGM2*, which suggested the change in protein expression might not result from altered gene expression but posttranslational regulation. ANPEP is the membrane-bound metalloproteases for general protein hydrolysis and also involved in cancerogenesis and tumor angiogenesis [18]. It is also recognized as CD13, the known cancer-stem cell marker for liver cancers [19].

**Table 2:** Top 10 enriched annotation clustering of proteins identities changed in melanoma cells under suspension.

**Category of annotation clustering (CC/BP/MF)**	**Number of proteins**	**Enrichment score**
**Blood vessel / vasculature development**	**6**	**2.19**
Protein localization	4	1.42
Pigment granule / Vesicle	5	1.40
Intracellular organelle lumen	13	1.14
Protein complex assembly	7	1.04
RNA processing	3	0.92
Negative regulation of catalytic activity	4	0.72
Metal ion transport	4	0.71
Vesicle-mediated transport	4	0.46
Positive regulation of signal transduction	3	0.44

**Table 3:** Top 10 enriched annotation clustering of proteins identities changed in melanoma cells reattached after suspension.

**Category of annotation clustering (CC / BP / MF)**	**Number of proteins**	**Enrichment score**
Intracellular organelle lumen	23	3.85
Macromolecular complex assembly	10	1.85
Focal adhesion	4	1.75
Chromosome organization	6	1.24
Nucleosome organization / DNA packaging	4	1.16
Metal ion binding	29	0.98
Membrane fraction	10	0.92
**Blood vessel / vasculature development**	**4**	**0.72**
Actin cytoskeleton binding	4	0.69
Lipid transport	4	0.59

In addition, we also noticed that integrin β4 was also downregulated in both suspended and re-attached melanoma cells. Although it was not indicated and highlighted in geneset of vessel formation by DAVID analysis, it was already known that integrin involved in different cell activities, including cell proliferation, adhesion, and migration [20], as well participated in several physiological or pathological conditions such as vessel formation or tumor angiogenesis through specific extracellular matrix protein recognition [21]. For instance, integrin α6β4 was recognized as a specific laminin-binding protein [22, 23]. Our previous study suggested melanoma cell suspension contributed to reduced laminin-binding ability through downregulation of SDC1 protein [1]. Our other research work had demonstrated the correlation of SDC1 with ANPEP expression that affected stemness characteristics of hepatoma cells [24]. We hypothesized that the regulation of ANPEP, SDC1, and integrin β4 expression might associate with loss of vasculogenic phenotype upon suspension of melanoma cells.

### Laminin-binding integrin beta4 was downregulated by melanoma cell suspension, SDC1 downregulation, and ANPEP downregulation

**Figure 3:**
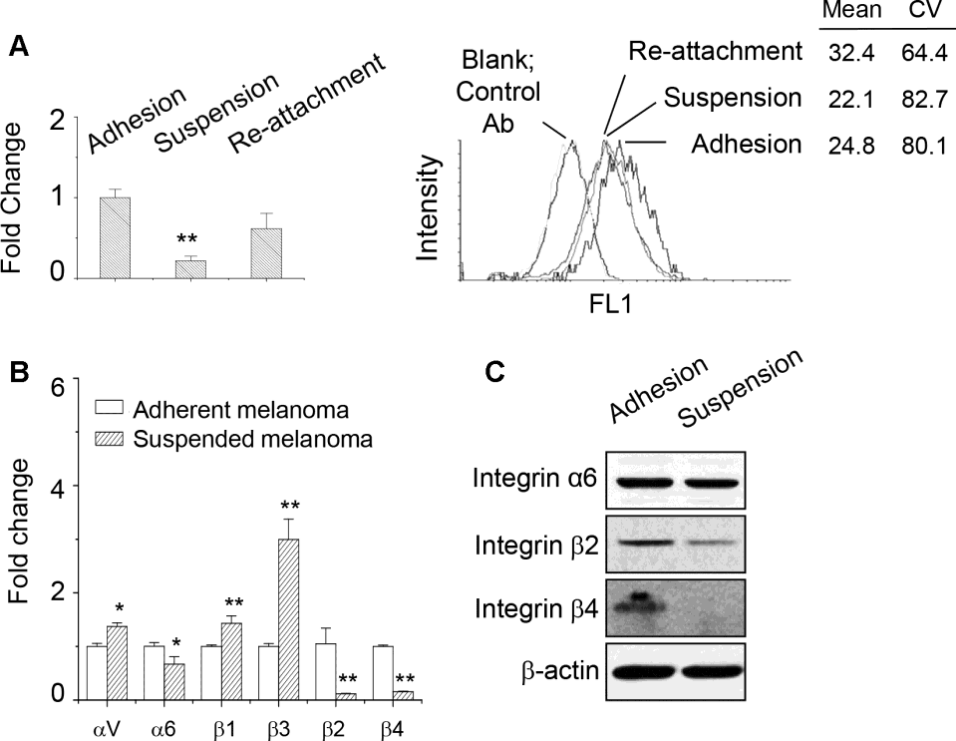
**ANPEP expression was downregulated upon cell suspension.** (**A**) The expression level of ANPEP in adherent, suspended, and re-attached melanoma cells as examined by qPCR and flow cytometry. Downregulation of ANPEP at the surface of suspended or re-attached melanoma cells was observed. Data were mean ±S.D. (n=3); **, *p* < 0.01. (**B**) Expression of integrin isoforms in adherent and suspended melanoma cells as examined by qPCR. Data were mean ±S.D. (n=3); *, *p* < 0.05; **, *p* < 0.01. (**C**) Integrin α6, β2, and β4 protein expression upon cell suspension as examined by western blot.

We examined the expression of ANPEP in adherent, suspended, and reattached melanoma cells by qPCR and flow cytometry. As seen in Figure 3A, ANPEP mRNA expression was significantly downregulated upon cell suspension, while partially restored by re-attachment after suspension. Expression at the cell surface was also examined using flow cytometry. As seen in Figure 3A, surface levels of ANPEP in suspended and re-attached melanoma cells were reduced, which were consistent with qPCR results.

Previously, we found that anchorage independence enabled the decreased SDC1 expression and altered the expressions of several integrin isoforms [1]. Consistent with our previous observation by microarray analysis, qPCR results also suggested that cell suspension upregulated integrin αV, β1, and β3; while integrin α6, β2, and β4 were downregulated (Figure 3B). This indicated the downregulation of integrin α6β4 would correlate with the reduced laminin-binding ability [1]. The protein expressions of integrin isoforms were also examined by western blot. As shown in Figure 3C, integrin β2 and β4 protein expression were reduced by cell suspension. However, integrin α6 protein level was not affected by cell suspension.

**Figure 4:**
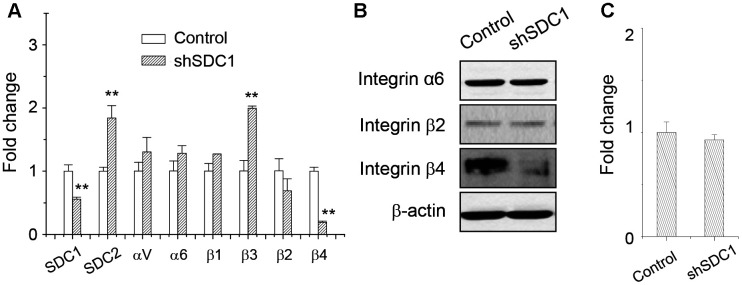
**Integrin β4 expression was downregulated upon suppression of SDC1 expression.** (**A**) Effect of SDC1 downregulation at expression of integrin isoforms as examined by qPCR. Data were mean ±S.D. (n=3); **, *p* < 0.01. (**B**) Integrin α6, β2, and β4 protein expression after SDC1 downregulation as examined by western blot. (**C**) SDC1 downregulation by shSDC1 did not change the level of ANPEP expression as examined by qPCR. Data were mean ±S.D. (n=3).

Since SDC1 level also affected the laminin-binding ability and it was downregulated in suspended melanoma, we checked whether SDC1 expression level would affect laminin-binding integrin expression. As seen in Figure 4A, the transfection of SDC1-specific shRNA suppressed SDC1 expression, but upregulated SDC2 expression, which was consistent with our previous observation [2]. Integrin β3 expression was upregulated, while integrin β2 expression was marginally reduced by SDC1-specific shRNA transfection. Only integrin β4 expression was significantly downregulated by SDC1-specific shRNA transfection. We suggested that integrin β4 expression would be specifically regulated by SDC1. The protein expressions of integrin isoforms were examined by western blot. As shown in Figure 4B, only integrin β4 protein expression was reduced by suppression of SDC1 expression. Although integrin β2 protein expression was reduced by cell suspension (Figure 3B and 3C), we suggested that integrin β2 expression would be regulated by other factors under anchorage-independence. In addition, SDC1 downregulation by shSDC1 did not change the level of ANPEP expression (Figure 4C). This implied that ANPEP would unidirectionally regulate SDC1 expression and sequentially affect the integrin expression.

**Figure 5:**
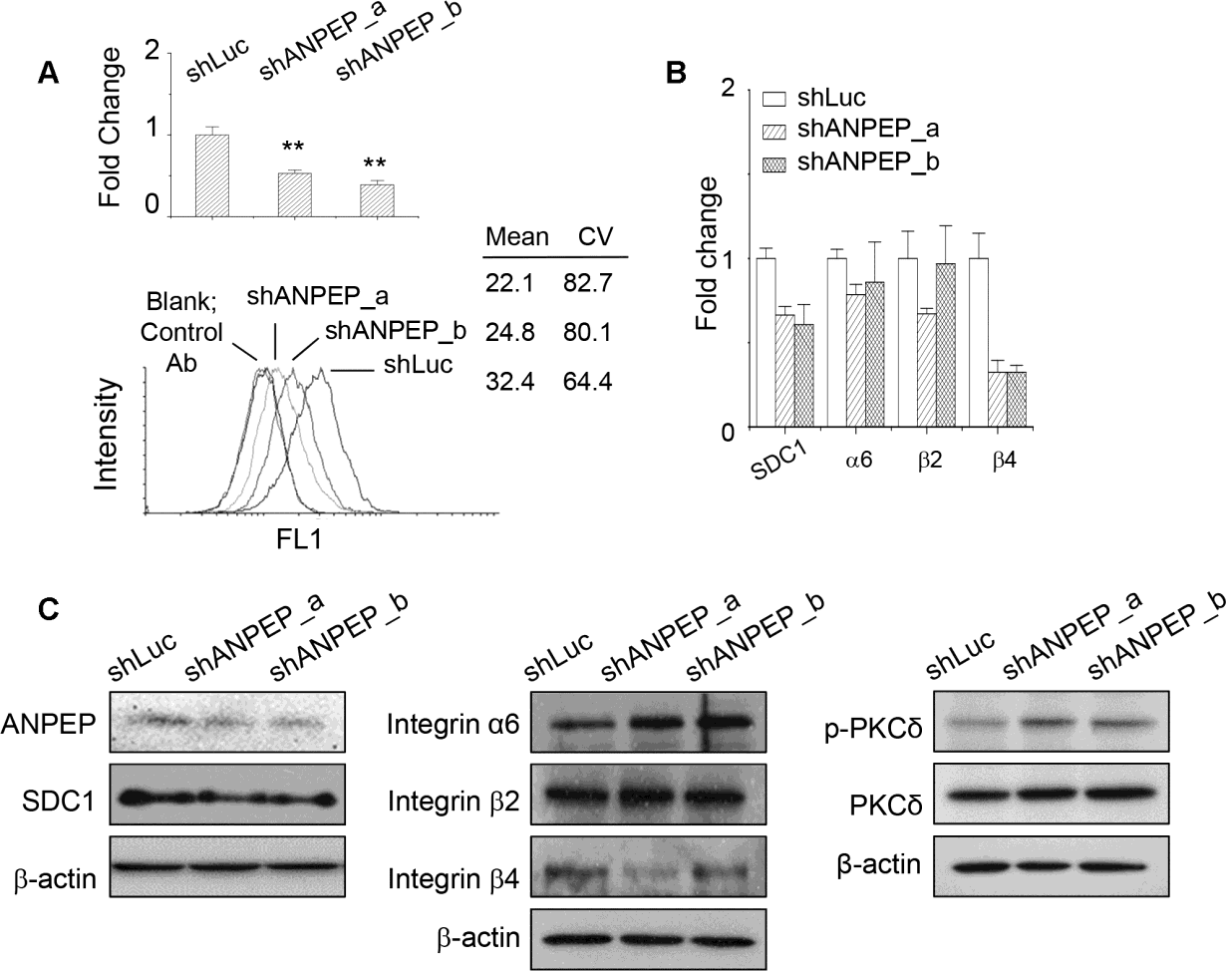
**ANPEP regulated SDC1 and integrin β4 expression through PKCδ phosphorylation.** (**A**) Suppression of ANPEP expression by ANPEP-specific shRNAs and were examined by qPCR and flow cytometry. Data were mean ±S.D. (n=3); **, *p* < 0.01. (**B**) Effect of ANPEP downregulation at expression of SDC1 and integrin isoforms as examined by qPCR. Data were mean ±S.D. (n=3); **, *p* < 0.01. (**C**) Effect of ANPEP downregulation at expression of integrin isoforms, SDC1, and PKCδ phosphorylation as examined by western blot.

In order to investigate whether ANPEP level in melanoma cells affected the expression of integrin isoforms and vascular phenotypes as we observed in suspended or reattached melanoma cells, we transfected ANPEP-specific shRNAs into melanoma cells. As seen in Figure 5A, shRNAs transfection reduced ANPEP expression levels (53% and 39% for shANPEP_a and shANPEP_b, respectively) in melanoma cells. The expression of ANPEP at the cell surface was also suppressed by shRNA transfection as evidenced by flow cytometry (Figure 5A). In addition, the expression levels of SDC1 and integrin isoforms upon suppression of ANPEP expression were examined by qPCR and western blot. As seen in Figure 5B, SDC1 and integrin β4, but not significant for integrin α6 and β2, were downregulated by suppression of ANPEP expression as examined by qPCR. As the protein expression, suppression of ANPEP protein expression reduced SDC1 protein expression (Figure 5C). The relative protein levels were reduced as 86% and 74% for shANPEP_a and shANPEP_b, respectively. Moreover, the protein level of integrin β4 was significantly reduced by ANPEP downregulation (Figure 5C), however, integrin α6 and β2 protein levels were increased and remained unchanged, respectively. Nevertheless, these indicated the expression axis ANPEP/SDC1/integrin β4 existed in melanoma cells, which was downregulated by anchorage independence. Previously, we had shown that the downregulation of SDC1 protein was mediated by the PKCδ activation in suspended melanoma cells [1]. So that we investigated whether ANPEP levels affected with PKCδ activation. As seen in Figure 5C, the levels of PKCδ phosphorylation were increased, while PKCδ protein expressions relatively remained unchanged upon the transfection of ANPEP-specific shRNAs. This suggested the regulatory axis of ANPEP/SDC1/integrin β4 that would be affected by the status of PKCδ activation/inactivation upon detachment stress.

### ANPEP downregulation in suspended melanoma conferred the characteristics of invasiveness loss but increased tumor growth

**Figure 6:**
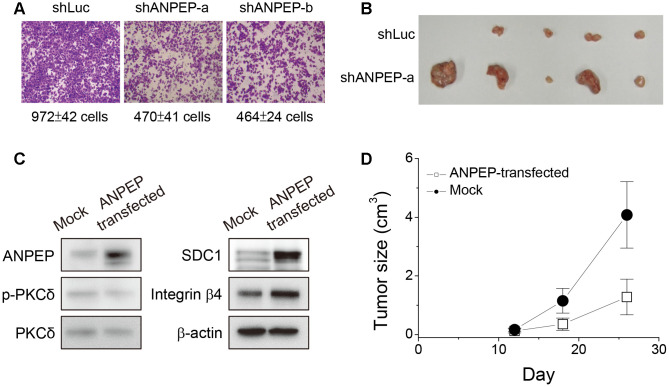
**ANPEP was tumor suppressive in melanoma cells.** (**A**) Suppression of ANPEP expression inhibited cell migration ability (**B**) Suppression of ANPEP expression promoted xenograft tumor growth as inoculated with adherent melanoma cells. (**C**) Effect of ANPEP overexpression at PKCδ phosphorylation, SDC1, and integrin β4 protein expression as examined by western blot. (**D**) ANPEP overexpression reduced the xenograft tumor formation as inoculated with suspended melanoma cells Data were mean ±S.D. (n=5).

In our previous study, anchorage-independence of melanoma cells by treatment of cell suspension contributed to the loss of cell invasiveness [1]. Since ANPEP expression was downregulated upon cell suspension, we examined the effects on ANPEP-specific shRNAs at cell migration ability. As seen in Figure 6A, cell migration ability was inhibited by transfection of ANPEP-specific shRNAs in the transwell migration assay. This was consistent with reported literatures that ANPEP played role in melanoma cell invasion [25, 26]. Next, we examined the effect of suppression in ANPEP expression at xenograft tumor growth. We subcutaneously inoculated melanoma cells transfected with control vector (shLuc) or ANPEP-specific shRNA (shANPEP-a). As shown in Figure 6B, the subcutaneous tumors derived from shANPEP-transfected melanoma cells were relatively larger than those derived from shLuc-transfected melanoma cells. This suggested downregulation of ANPEP in suspended melanoma cells would promote tumor malignancy. This was consistent with our previous study that anchorage-independence of melanoma cells by cell suspension promoted tumor growth [3].

Other literatures suggested ANPEP was a therapeutic target or prognostic marker of other tumors, that indicated its role in tumor malignancy [18, 19, 25]. However, we observed that the reduced level of ANPEP promoted melanoma tumor growth. In order to reconfirm this result, we overexpress ANPEP protein and examined its effects in protein expressions of ANPEP/SDC1/integrin β4 axis and xenograft tumor growth. As seen in Figure 6C, overexpression of ANPEP reduced the extent of phosphor-PKCδ, but increased the expression of SDC1 and integrin β4. This strengthened our conclusion that ANPEP would regulate the downstream expression of SDC1 and integrin β4 protein. Detachment treatment of melanoma cells would promote extensive xenograft tumor growth (Figure 6B). However, ANPEP overexpression reduced suspended cell-inoculated tumor growth (Figure 6D). This suggested ANPEP might play a role as tumor suppressor in melanoma cells under detachment stress.

### ANPEP downregulation contributed to the downregulation of SDC1 and integrin β4 *in situ*

**Figure 7:**
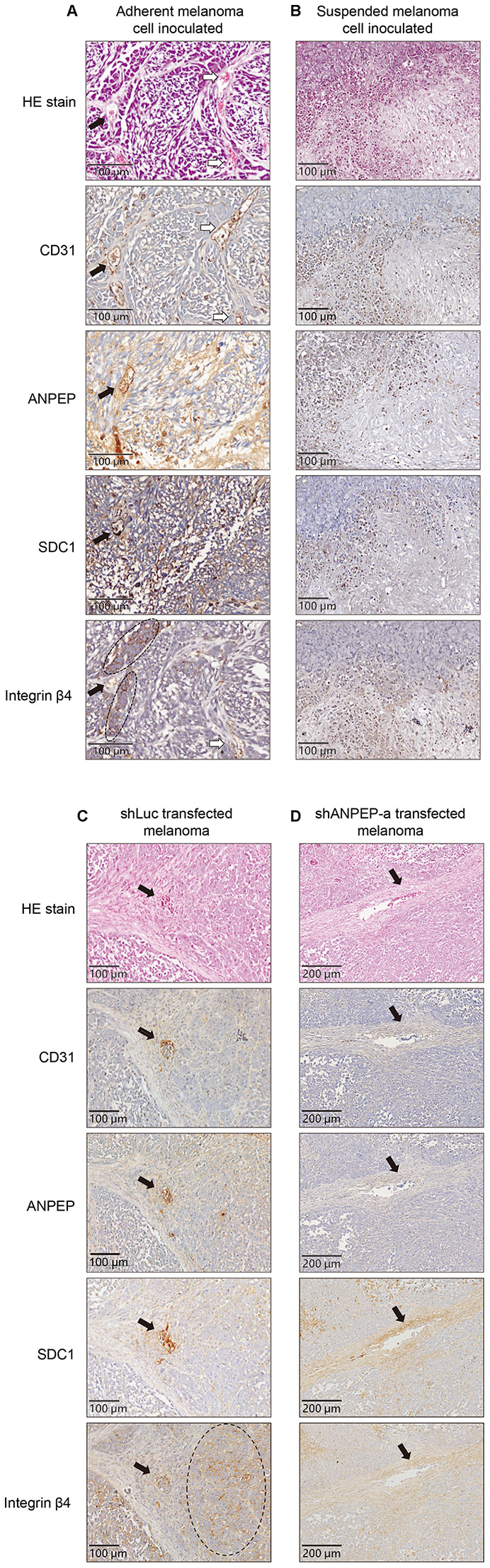
**Immunostaining showed different vascular phenotypes with different protein expressions upon cell suspension and suppression of ANPEP expression.** Photomagnification of tumor tissues derived from (**A**) adherent melanoma cells or (**C**) shLuc-transfected cells with HE stain and immunostaining of CD31 showed vascular structures. Positive ANPEP, SDC1, and integrin β4 expression in these tumor tissues were also presented. Filled arrow, vascular cavities with integrin β4-positive cells-surrounded. Empty arrow, vascular cavities with integrin β4-positive cells. No vascular structures with CD31/ANPEP/SDC1/integrin β4 expression were seen in those from (**B**) suspended melanoma cells or (**D**) shANPEP-transfected cells. The arrows indicated the locations of RBC-infiltrated cavities.

It was known that integrin proteins involved in different cell activities, including cell proliferation, adhesion, and migration [20], as well participated in several physiological or pathological conditions such as vessel formation or tumor angiogenesis [21]. We further examined whether altered expressions of ANPEP/SDC1/integrin β4 were associated with different vascular phenotypes *in vivo*. As seen in Figure 7A, HE stain and immunostaining of CD31 showed vascular structures in the tumor derived from adherent melanoma cells. It also showed positive ANPEP, SDC1, and integrin β4 expression around these vascular cavities. Interestingly, Expression of integrin β4 showed different sub-phenotypes. The filled arrow indicated the location of vascular cavities. Some integrin β4-positive cells (dashed eclipses) were surrounded these cavities, while some integrin β4 positive cells were lined into vascular cavities as the same in CD31-positive cavities (the locations were indicated by empty arrows). We suggested these might be the trace of trans-differentiation for integrin β4-positive cells to become vascular structures. For the tumor derived from suspended melanoma cells, no vascular structure was observed by HE stain. No cavities with positive CD31, ANPEP, SDC1, or integrin β4 immunostaining was seen.

In the tumor sample inoculated with shLuc-transfected cells, several vascular cavities were seen with positive CD31, ANPEP, SDC1, and integrin β4 immunostainings (Figure 7B). The large areas of integrin β4-positive cells were also observed (dashed ellipse) and surrounded at the fibrous structure. In the tumor sample inoculated with shANPEP-a transfected cells, we still could find some vascular cavities with RBCs (empty arrow). However, no CD31-positive or ANPEP-positive cells to form these vascular cavities, while weak SDC1 and integrin β4 immunostaining were seen. These cavities seem to be formed by loose connective tissues with diluted fibrous structures, which enabled the RBC diffusion. Nevertheless, we suggested the ANPEP expression correlated with SDC1/ integrin β4 expression, which affects vasculogenic phenotypes to show CD31-positive vascular cavities.

## DISCUSSION

Tumor metastasis is a complicated process mediated by many molecular mechanisms. Of them, detachment from their original location is the initial step of tumor dissemination. We simulated the status of melanoma cells that suffered from detachment stress by suspension cell culture. The detachment stress altered the phenotype of cell invasiveness [1], promoted tumor malignancy [3], and enhanced chemosensitivity [2] of melanoma cells. Those pieces of evidence suggested anchorage-independence of malignant melanoma would alter gene/protein expression through several key proteins and pathways. We thus were interested in whether anchorage independence would promote tumor heterogeneity *in situ*. In this paper, we adapted proteomic analysis on the protein contents from adherent, suspended, and reattached melanoma cells. We found that ANPEP was downregulated by cell suspension and its downregulation further suppressed the expression of SDC1 and integrin β4 that led to the loss of vascular structure in melanoma tumor.

ANPEP is a metalloenzyme for peptide cleavages. It plays roles in cell migration, invasion, angiogenesis, and tumor metastasis. They showed that ANPEP was upregulated in endothelial tumor cells and expressed in blood vessels undergoing angiogenesis [27]. A study using ANPEP inhibitor also showed its functional role in vasculogenesis [28]. The upregulation of ANPEP in blood vessels of inflammatory and neoplastic tissues was observed [29]. It is also recognized as CD13 as a marker for semiquiescent CSCs in human liver cancer cell lines [19]. Our previous microarray data (GSE42876 and GSE61671) showed downregulation of *ANPEP* gene expression in suspended melanoma cells and reattached melanoma cells.

SDC1 was showed to associate with the formation of blood vessels and tumor growth. It was shown that SDC1 promotes tumor growth and angiogenesis in endometrial cancer cells. It was associated with activation of MMP2 and MMP9 for the development of angiogenesis and invasive phenotype [9]. The knockdown of SDC1 in myeloma cells resulted in smaller and fewer tumor formation subcutaneously or intravenously, with impaired development of blood vessels [8]. SDC1 was expressed in the vascular endothelium during microvessel outgrowth through the regulation of αvβ3 and αvβ5 integrin activation [30]. Several literatures also suggested the interaction of SDC1 with integrin α6β4. Interaction of SDC1 and α6β4 integrin cytoplasmic domains would regulate ErbB2-mediated integrin activation in human squamous carcinoma cells [31]. Interaction of SDC1 with cytoplasmic domain of β4 integrin directed the coupling of integrin to human epidermal growth factor receptor-2, which affected cell survival and migration on laminin surface [32].

Previously, we demonstrated that anchorage-independence of melanoma cells impaired their laminin-binding ability through downregulation of SDC1 [1]. Of all the laminin-associated integrin isoforms [11], integrin α6β4 was shown to interact with SDC1 and involved in angiogenesis. Deletion of the signaling domain of the integrin β4 subunit reduced angiogenic response to bFGF. It was associated with α6β4 signaling-induced endothelial cell migration [33]. β4 signaling promotes both angiogenesis and tumorigenesis through the combination of multiple receptor tyrosine kinases, including ErbB2, EGF-R and Met [34].

It’s interesting that ANPEP downregulation reduced cell growth and cell invasiveness but resulted in elevated xenograft tumor growth. We explained that the reduced cell growth *in vitro* but enhance xenograft tumor growth *in vivo* would be derived from IL-8 upregulation. Our previous studies showed suspended melanoma cells had reduced SDC1 expression [1] but exhibited higher potential at xenograft tumor growth through elevated IL-8 levels [3]. We examined and observed the increased expression level of IL-8 after shANPEP-a transfection (data not shown). Our observation connects our previous works and reveals that downregulation of ANPEP in suspended melanoma cells regulates the ANPEP/SDC1/beta4- integrin axis.

The lack of vascular structures in suspended melanoma-derived tumors might be the loss of angiogenic heterogeneity, but not tumor lymphangiogenesis. Tumor lymphangiogenesis is another form of vasculogenic phenotype to support tumor growth [35]. The intratumoral lymphatics was significantly more in metastatic melanomas and correlated with poor prognosis [36]. Lymphatic vessel endothelial hyaluronan receptor 1 (LYVE1) is typical marker for lymphangiogenesis, but our microarray results showed no difference in LYVE1 expression.

**Figure 8:**
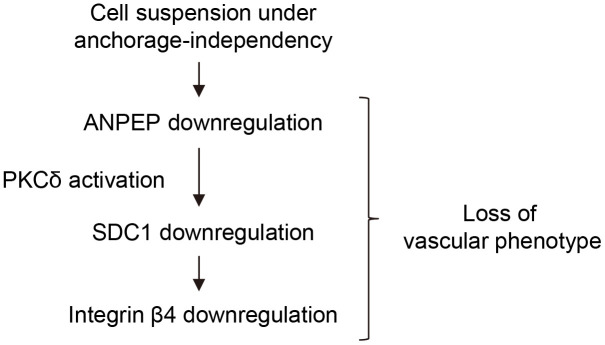
**Scheme for ANPEP-mediated downregulation of SDC1 and integrin β4 mediated by PKCδ activation, so that contributed to the loss of vasculogenic phenotypes.**

Although it was demonstrated that ANPEP, SDC1, and integrin β4 were associated with vessel formation/angiogenesis, our results suggested the sequential expression of ANPEP/SDC1/integrin β4 through PKCδ activation determined the vasculogenic phenotype of melanoma cells (Figure 8). Coupling with our previous papers [1–3], we suggested the detachment stress was the priming factor to be responsible for melanoma dormancy, malignancy, chemosensitivity, and altered phenotypes.

## MATERIALS AND METHODS

### Cell culture and cell transfection

Human melanoma A375 cells were purchased from Bioresource Collection and Research Center (BCRC; Hsinchu, Taiwan) with authentication. Adherent culture were maintained using culture dish (Corning Incorporated Life Sciences, Tewksbury, Massachusetts, USA) and DMEM medium supplemented with 10% (v/v) fetal bovine serum (Biological Industries Ltd., Cromwell, Connecticut, USA). Suspended melanoma cells were established and cultured by impaired attachment of adherent melanoma cells according to the reported procedures [37, 38]. Re-attached melanoma cells were established by seeding suspended cells onto culture dish and keeping at incubator for 1 day.

The plasmids expressing shRNAs against *ANPEP* (clone ID: TRCN0000050239 and TRCN0000050238) or *SDC1* (clone ID: TRCN0000072581) gene were purchased from the National RNAi Core Facility located at Institute of Molecular Biology/Genomic Research Center, Academia Sinica, Taiwan. The gene construct to overexpress ANPEP and the control vector were all purchased from Sino Biological (Beijing, China). Cells were transfected using TurboFect transfection reagent (Thermo Fisher Scientific Inc., Pittsburgh, Pennsylvania, USA) according to the manufacturer’s instruction. The transfected cells were selected and enriched under growth medium containing 5 μg/mL puromycin (for shRNA-transfect cells) or 0.5 mg/mL hygromycin B (for ANPEP-transfected cells).

### Animal experiment and IHC analysis

The animal experiments were supervised and regulated by Experimental Animal Care and Use Committee in FuJen Catholic University (permission number A9830). The nude mice (Nu/Nu strain, male, n=5) in 3 weeks old were inoculated intravenously (at least 3 cm from the root of tail) or subcutaneously (at both left and right abdomens) according to the experimental designs. Basically, 1x10^6^ adherent or suspended melanoma cells (in 200 uL of serum-free DMEM) was injected, and the mice were supplemented with free access to food and water in a specific pathogen free environment for 2 months. After animal scarification, the tumor tissues were harvested and fixed in 10% (v/v) buffered formalin. The tissues were then dehydrated in graded ethanol, and embedded in paraffin. The tissues in 3 μm thick sections were generated and stained with hematoxylin and eosin (HE stain) or immunohistochemical staining. The tissues were blocked and then incubated with desired antibodies. The monoclonal antibody (clone 3D8) against ANPEP protein was from Santa Cruz Biotechnology, Inc., Dallas, TX, USA. The primary antibodies against CD31 were from Bioss antibodies Inc. (Woburn, Massachusetts, USA). The primary antibodies against integrin β4 were from eBioscience Inc., San Diego, California, USA. The monoclonal antibody (clone B-A38) against SDC1 was from Novus Biologicals LLC., Littleton, CO, USA. Tissue-bound primary antibody was then visualized with a horseradish peroxidase-conjugated secondary antibody-3,3’-diaminobenzidine system (Rabbit PolyDetector HRP/DAB system; Spring Bioscience, Pleasanton, California, USA). Hematoxylin was used as a counterstain. These slides were scanned by digital microscope PreciPoint M8 (Precipoint, Inc., Freising, Germany). The images were captured by software viewpoint (Precipoint, Inc., Freising, Germany).

### Quantitative proteomic analysis of protein contents by dimethyl labeling approach

The protein contents in adherent, suspended, and re-attached melanoma cells were quantified using dimethyl peptide labeling approach. The process of protein digestion, dimethyl peptide labeling, fractionation, LC-MS/MS analysis, and data analysis were done according to the published paper [39]. The cell lysis and protein extraction were separated into cytoplasmic and nuclear fractions using NE-PER™ Nuclear and Cytoplasmic Extraction kit (Thermo Fisher Scientific Inc., Pittsburgh, Pennsylvania, USA). Protein lysates were lyophilized and resuspended in 100 mM triethylammonium bicarbonate and 8M urea. The proteins were then reduced with 10 mM dithiothreitol at room temperature for 30 min and *S*-alkylated with 50 mM iodoacetamide at room temperature in the dark for 30 min. The resulting proteins were sequentially digested with endopeptidase Lys-C (1:100 w/w) (WAKO, Tokyo, Japan) at room temperature for 2 h, and further digested with sequencing-grade modified trypsin (1:100 w/w) (Promega corporation, Madison, Wisconsin, USA) at room temperature overnight.

Digested peptides from suspended or re-attached melanoma cells were labeled with isotopic formaldehyde (^13^CD_2_O, heavy-labeled); while those from adherent melanoma cells were labeled with formaldehyde (CH_2_O, light-labeled). After dimethyl labeling, equal amounts of the heavy- and light-labeled peptides were mixed and desalted. To increase peptide identification, the dimethyl-labeled peptides were further separated into 5 fractions using a strong cation exchange StageTip (Empore Cation Exchange-SR membrane; 3M Company, St. Paul, Minnesota, USA) by elution with different salt concentrations (30, 60, 90, 200, and 500 mM ammonium acetate in 15% acetonitrile and 0.1% trifluoroacetic acid). The eluted peptides were desalted, vacuum-dried, and resuspended in 2% acetonitrile and 0.5% acetic acid for mass analysis.

The fractionated peptides were analyzed using a nanoflow LC-MS/MS on a Dionex Ultimate 3000 RSLC nanosystem online-coupled to an LTQ Orbitrap XL mass spectrometer (Thermo Fisher Scientific Inc., Pittsburgh, Pennsylvania, USA). The peptides were loaded onto a 100 μm × 15 cm tip column with 3-μm ReproSil-Pur 120 C18-AQ reverse-phase beads (Dr. Maisch HPLC GmbH, Ammerbuch-Entringen, Germany). The peptides were eluted at a flow rate of 500 nL/min by gradient elution with two mobile phases: 0.5% acetic acid in water (solution A) and a mixture of 0.5% acetic acid and 80% acetonitrile (solution B). The gradient was 5–10% B (5 min), 10–40% B (60 min), 40–100% B (5 min), and 100% B (10 min). The LTQ Orbitrap XL mass spectrometer was operated in a data-dependent mode with a spray voltage of 2400 V. The full MS scan spectra (m/z 300–1600) were acquired with a solution of 60,000 at m/z 400 in the Orbitrap analyzer with auto gain control value of 5×10^5^. The top 10 most intense precursor ions with charge state ≥ +2 were sequentially isolated with an isolation width of 2 Da and auto gain control value of 1×10^4^. The selected precursor ions were then fragmented using collision-induced dissociation in the linear ion trap with 35% normalized collision energy. The activation q was set to 0.25, and the activation time was set to 30 ms. Dynamic exclusion was set at 90 s with an exclusion list size of 500. A lock mass of m/z 445.120024 was applied for all LC-MS/MS analysis.

Raw files from LC-MS/MS were analyzed using MaxQuant software (version 1.6.1.0). The peak lists were searched with SWISS-PROT sequence database. Cysteine carbamidomethylation was set as a fixed modification. Variable modifications were the *N*-acetylation of proteins and oxidation of methionine. The search setting included minimum peptide length of 7 amino acids, enzyme specificity with a maximum of 2 missed cleavages, peptide tolerance of 4.5 ppm, MS/MS tolerance of ±0.5 Da, and false discovery rates (FDRs) at 1%. The results from adherent melanoma cells were as control, and those from suspended or re-attached melanoma cells were compared. The potential upregulated or downregulated protein targets were initially filtered with Perseus software (version 1.6.2.3) [40]. The significant cut-off thresholds were based on the Benjamini–Hochberg FDR calculation with threshold value *p* < 0.05 [41]. The mass spectrometry proteomics data have been deposited to the ProteomeXchange Consortium via the PRIDE partner repository with the dataset identifier PXD011883.

The protein identities with significantly upregulated or downregulated in melanoma cells under suspension (or reattachment upon suspension) were functionally annotated using DAVID v6.7 (The Database for Annotation, Visualization and Integrated Discovery [16, 17]). The functional annotation clustering of these protein identities was done with high classification stringency and the parameters of kappa similarity term overlap 3, similarity threshold 0.85, classification with initial and final group membership 3, multiple linkage thresholds 0.50, enrichment thresholds 1.0 and *p*-value < 0.05.

### Polymerase chain reactions and statistical analysis

The levels of mRNA in cultured cells were analyzed by qPCR. The cDNAs were synthesized by MMLV HP reverse transcriptase (Epicentre Inc., Madison, Wisconsin, USA) using freshly prepared RNA as PCR template. Quantitative real-time PCR was performed using VeriQuest Fast SYBR green qPCR reagent (Affymetrix Inc., Santa Clara, California, USA) in a StepOne Plus real-time PCR system (Thermo Fisher Scientific Inc., Pittsburgh, Pennsylvania, USA). The 2^-ΔΔCT^ method was used to determine the relative gene expression using GAPDH as control. The *p*-value of < 0.05 or < 0.01 was statistically significant and was indicated in each figure. The forward and reverse primers used were: ANPEP, gccgtgtgca caatcatcgcact and caccagggagcccttgaggtg; SDC1, gctctggggatgactctgac and gtattctcccccgaggtttc; SDC2, ccagccgaagaggatacaaa and gcgttctccaaggtcatagc; Integrin αV, agatctggaccaggatggtt and atctgtggctcctttcattg; Integrin α6, caagatggctacccagatat and ctgaatctgagagggaacca; Integrin β1, gttacacggctgctggtgtt and ctactgctgacttagggatc; Integrin β2, tgcgtcctctctcaggagtg and ggtccatgatgtcgtcagcc; Integrin β3, accactgatgccaagactca and gcatcaacaatgagctggag; Integrin β4, aacgatgaacggtgccacct and ctccacgatgttggacgagt; GAPDH, gagtcaacggatttggtcgt and gatctcgctcctggaagatg.

### Western blot and antibodies

To harvest cell lysate for western blot analysis, cells were washed and disrupted by lysis buffer (10 mM Tris-HCl, 5 mM EDTA, pH 8.0, 1% TritonX-100, and protease inhibitors) and kept on ice for 30 min. The lysate was then centrifuged at maximum speed using a desktop centrifuge at 4^o^C for 10 min. Protein concentrations were quantified by protein assay kit (Bio-Rad Laboratories Inc., Hercules, California, USA). For the characterization of SDC1 protein, a pretreatment to remove glycosaminoglycan chain by digestion enzymes (all purchased from Sigma-Aldrich China, Inc., Shanghai, PR China) was done. 50 μg cell lysate was pretreated with 0.83 mIU heparinase I, 0.83 mIU heparinase II, 0.83 mIU heparinase III, and 0.83 mIU chondroitinase in 200 uL reaction buffer (20 mM Tris-HCl, pH 7.5, 4 mM CaCl_2_, and 0.1 % (w/v) BSA) at 37 ^o^C for 16 hr. The proteins were precipitated by trichloroacetic acid and solubilized by solubilizing solution (10 mM Tris-HCl, pH8.0, 5 mM EDTA, and 2 M urea) then subjected to SDS-PAGE.

Western blot was performed according to standard protocol. Briefly, the protein mixture was subjected to SDS-PAGE and transferred onto a PVDF membrane followed by blocking with 5% (w/v) skim-milk. The membrane was then incubated in primary antibodies (1:1000 in 5% skim-milk in TBST) for 2 hr at room temperature, and HRP-conjugated secondary antibody (1:20000) for 1 hr at room temperature followed by enhanced chemiluminescent (Millipore Co., Massachusetts, USA) detection. The primary antibodies against ANPEP, integrin β4, SDC1 were the same as used in IHC. The primary antibodies against integrin α6 and integrin β2 were from Bioss antibodies Inc. (Woburn, Massachusetts, USA). The primary antibodies against PKCα, phospho-PKCα, PKCδ, and phospho-PKCδ were purchased from Cell signaling Inc., Danvers, Massachusetts, USA. The primary antibody against β-actin was purchased from GeneTex Inc., Hsinchu, Taiwan.

### Flow cytometry analysis

ANPEP expression analyzed by flow cytometry was performed accordingly [1]. The 1 x 10^5^ cells were incubated with 1 μg anti-ANPEP primary antibody (3D8; Santa Cruz Biotechnology, Inc., Dallas, Texas, USA) overnight at 4^o^C, and 0.5 μg FITC-labeled secondary antibody at room temperature for 1 hr. The labeled cells were then analyzed by Partec flow cytometer ML (Partec North America, Inc., Swedesboro, New Jersey, USA).

### Transwell migration assay

For transwell migration assay, culture insert (8 um pore size; BD bioscience, Inc., San Jose, California, USA) were precoated with matrigel (BD bioscience, Inc., San Jose, California, USA). 1x10^5^ cells (in DMEM medium with 1% (v/v) fetal bovine serum) were applied to the insert. The bottom wells contained 600 uL of culture medium with 10% (v/v) fetal bovine serum and were left un-agitated in the cell incubator. After 24 hrs, the cells attached to the transwell membrane were fixed with 0.5% (w/v) crystal violet and washed extensively. The stained cells were visualized and counted under microscopy.
